# Factors associated with mpox awareness among men who have sex with men recruited through the internet: a cross-sectional survey in China

**DOI:** 10.3389/fpubh.2025.1594225

**Published:** 2025-06-26

**Authors:** Yun Fu, Weiyong Chen, Rui Yuan, Xing Wang, Zhongrong Yang

**Affiliations:** ^1^Huzhou Center for Disease Control and Prevention, Huzhou, China; ^2^Department of HIV/STD Control and Prevention, Zhejiang Provincial Center for Disease Control and Prevention, Hangzhou, China; ^3^Huzhou Third Municipal Hospital, The Affiliated Hospital of Huzhou University, Huzhou, China

**Keywords:** associated factors, infection, internet, monkeypox (mpox), men who have sex with men, risk

## Abstract

**Objective:**

This study aimed to provide a scientific basis for formulating mpox prevention and control strategies for men who have sex with men (MSM) by analyzing the associated factors.

**Methods:**

We conducted online surveys with individuals recruited through the Internet in May 2024. Univariate and multivariate logistic regression analyses were used to identify factors influencing knowledge about mpox.

**Results:**

In the 604 participants surveyed, the mpox knowledge rate was 62.91%. Multivariate logistic regression analysis results showed that the following factors were associated with higher levels of mpox knowledge (*p* < 0.05): age 30 years and above, education level of college or above, average monthly income ≤6,000 CYN, belief that using rush poppers increases the risk of HIV infection, recent exposure to HIV or mpox information through online media, and recent receipt of HIV or mpox prevention services.

**Conclusion:**

Mpox-prevention knowledge should be promoted among individuals who have lower education levels, while the dissemination of information on HIV and mpox through online media should be increased to reduce the risk of mpox or HIV infection among this population.

## 1 Introduction

Mpox is a zoonotic disease caused by infection with the mpox virus and poses a significant threat to public health (1). The virus can spread through contact with infected animals, direct contact with infected individuals, or respiratory droplets, as well as vertical transmission from mother to child (2). The mortality rate among individuals infected with the mpox virus ranges from approximately 1–10% (3), while those with weakened immune systems are at an increased risk of severe complications and mortality (4). Before 2003, mpox cases were predominantly clustered in the tropical rainforests of Central and West Africa. A global outbreak of mpox emerged in 2022, with a notable prevalence among men who have sex with men (MSM) (5). On July 23, 2022, the World Health Organization (WHO) officially recognized the mpox outbreak as a “public health emergency of international concern” (6). The outbreak was successfully contained through coordinated international efforts, including the declaration of a mpox emergency by the WHO in May 2023. In September 2023, a genetically distinct strain of the mpox virus, designated clade 1b, was detected in the Democratic Republic of the Congo, which fueled sustained community transmission and held potential for cross-border spread beyond Africa. Current evidence suggests that symptoms induced by clade 1 variants may be more severe than those induced by clade 2 strains, implying an elevated risk of severe illness and mortality. Subsequently, on August 14, 2024, the WHO issued a declaration labeling the mpox outbreak as a “public health emergency of international concern.” This signified for the second time, following the initial proclamation on July 23, 2022, that mpox poses a significant global health threat.

Both mpox and HIV can be transmitted through sexual activity, with mpox primarily spreading through direct contact with secretions or exudates from lesions of infected individuals during sexual encounters (7, 8). MSM have an increased risk of mpox infection (9); however, significant disparities exist in their knowledge and awareness of mpox prevention. In the MSM population, some individuals may lack precise understanding of mpox, leading to inadequate preventive measures. Therefore, it is imperative to prioritize health education and disease prevention awareness among this population. As mobile internet technology continues to advance and gain popularity, diverse information can be accessed conveniently (10). MSM often demonstrate a competent level of online engagement and information retrieval skills, rendering them more open to health promotion and education campaigns delivered through the internet (11, 12). Consequently, implementing health education targeted at the MSM population, specifically addressing the significant health concerns of mpox, and leveraging Internet resources for recruitment and promotion in a timely manner would significantly enhance preventive efforts.

Customized promotional strategies and educational initiatives tailored to specific groups are being implemented by conducting internet-based assessments of awareness and cognitive levels regarding mpox prevention among the MSM population (13). This individualized approach aims to improve the awareness of mpox prevention among the MSM population, facilitating the implementation of deep-rooted health education programs. When examining awareness levels and factors related to mpox prevention in the MSM community, the various influencing factors must be comprehensively considered. In addition to individual health awareness and educational background, aspects such as personal income and sexual behavior characteristics significantly influence awareness levels (14, 15). A thorough analysis of these key factors is essential for customizing health education efforts and advancing mpox prevention initiatives effectively. Nonetheless, the current understanding of mpox prevention and control knowledge among MSM who are recruited online remains uncertain. Thus, this study recruited MSM online and conducted surveys to assess their awareness of mpox prevention knowledge and its influencing factors, laying a scientific foundation for developing tailored strategies for mpox prevention and control in this population.

## 2 Materials and methods

### 2.1 Study design and ethical declaration

This cross-sectional survey was conducted through online recruitment of MSM by a non-governmental organization in May 2024, and the survey was conducted by surveyors and not done by respondents themselves. The research protocol was approved by the Ethics Committee of the Huzhou Center for Disease Control and Prevention (Approval No: HZ2023003), and all participants provided informed consent before proceeding to the questionnaire.

### 2.2 Participants

The study participants were MSM recruited via the Internet. Questionnaires were completed via an online platform (such as questionnaire surveys, exams, assessments, voting, etc.) named Wenjuanxing, after ensuring that each WeChat account could complete the survey only once. This study utilized internet recruitment rather than random sampling, thereby we employed a non-probability sampling method akin to snowball sampling (16). To determine the sample size, the following calculation method was applied: utilizing data from behavioral monitoring obtained in repeated surveys, we derived the formula *N* = 400 × *Q*/*P*. Here, P represents the estimated proportion of the relevant behavior occurring during the survey; specifically, it is based on the proportion of MSM who reported unprotected anal intercourse, estimated between 28.7 and 53.0% according to related surveys (17–19). In this study, we used *p* = 47.5%, *Q* = 1 − *P*. Given these parameters, the study necessitates a minimum of 442 participants recruited via the internet.

The inclusion criteria were age ≥18 years, have engaged in sexual activities between males (including masturbation, oral sex, and anal sex), and willingness to participate in the survey after providing informed consent. We excluded participants who were aged <18 years, who recruited sexual partners offline or at fixed locations, who were unwilling to participate after having provided informed consent, or had mental or cognitive disorders based on self-reporting.

A total of 626 MSM completed the survey questionnaire, with 604 providing informed consent and completing the survey, and 22 declining to participate.

### 2.3 Survey content

Our questionnaire design was primarily based on the Chinese MSM sentinel monitoring and college student survey questionnaires (20–22). After conducting a presurvey and improving the questionnaire variable settings, a formal survey was conducted. The questionnaire included general sociodemographic characteristics (including age, educational level, and average monthly income), characteristics related to sexual behavior (sexual attitudes, condom use during sexual activity, whether there is currently a regular sexual partner, whether participated in sexual activity in the past 6 months, and whether alcohol was consumed before engaging in sexual activity with the same sex), whether HIV prevention services have been received in the past year (educational materials and condom distribution, counseling services, and training lectures), whether there is concern about contracting HIV through same sex, whether an HIV test has been taken in the past year, self-efficacy regarding condom use, and whether pre-exposure prophylaxis for HIV has been used in the past 6 months (16).

### 2.4 Definition of related indicators

Definition of awareness of mpox knowledge included five multiple-choice questions regarding the source of infection, transmission routes, clinical symptoms, susceptible populations, and preventive measures. Each question had correct and “do not know” options. Answering at least one correct option for each question was considered a correct response, and answering all five questions correctly indicated awareness of mpox prevention. The participants were categorized into aware or unaware groups based on their knowledge of mpox prevention.

Definition of receipt of prevention services refers to whether individuals have received HIV or mpox-related informational materials, distributed condoms, counseling services, training workshops, in the past year. A one-night stand was defined as a temporary sexual encounter between MSM.

### 2.5 Quality control

Effective quality control measures were implemented through questionnaire restrictions, such as mandatory questions, logical skips, and response range limitations, to obtain valid questionnaires that met the recruitment criteria. The surveys were conducted by surveyors, and the surveyors underwent standardized training before conducting the survey using the uniform questionnaire. Prior to the survey, researchers explained the purpose, significance, methodology, and privacy policy to the participants, including this information in the introductory section of the questionnaire. Participants were informed that the survey aimed to develop HIV and sexually transmitted infection-prevention strategies for MSM, emphasizing the anonymity of the survey and the analysis of group data, rather than individual data.

### 2.6 Statistical analysis

The software programs used for analyses included R v.4.3.2^1^ and SPSS software (version 19.0, IBM SPSS Inc.; Armonk, NY, USA). Continuous data are presented as means ± standard deviation. Categorical data are expressed as frequencies or rates and were analyzed using the chi-squared test. The dependent variable was participants’ awareness of mpox prevention (aware = 1, unaware = 0). Variables with *p* < 0.10 in the univariate regression analysis were included as independent variables in the model for multivariate logistic regression analysis using the enter method. Statistical significance was defined by *p* ≤ 0.05.

## 3 Results

### 3.1 General demographic characteristics

A total of 604 participants recruited for this study had an average age 28.04 ± 6.43 years. The youngest participant was 19 years old and the oldest was 53 years old, with the 30–53 year old group accounting for 30.46% (184/604) of the participants. Unmarried and divorced individuals accounted for 93.05% (562/604). Participants with a rural household registration constituted 38.58% of the sample (233/604). Those with a college education or above comprised 77.48% (468/604) of the sample, and currently enrolled students constituted 20.53% (124/604) of the sample. Individuals with a monthly income ≤6,000 CNY accounted for 58.61% (354/604) of the sample. Harmonious family relationships were reported by 54.80% (331/604) of participants. Table 1 summarizes the results of the study.

**Table 1:** Associated factors of mpox awareness among participants.

Variables	Mpox awareness group (*n* = 380)		Mpox unawareness group (*n* = 224)		Univariate analysis		Multivariate analysis	
	*n*	%	*n*	%	OR (95%CI)	*p*	OR (95%CI)	*p*
Age (yrs)								
19–29	251	59.8	169	40.2	Ref.		Ref.	
30–53	129	70.1	55	29.9	1.58 (1.09–2.30)	0.016	1.65 (1.10–2.51)	0.016
Marital status								
In marriage or cohabitation	29	69.0	13	31.0	Ref.			
Unmarried or divorced	351	62.5	211	37.5	0.75 (0.37–1.44)	0.395		
Residence registration								
Urban areas	230	62.0	141	38.0	Ref.			
Rural areas	150	64.4	83	35.6	1.11 (0.79–1.56)	0.445		
Educational level								
High school or below	77	56.6	59	43.4	Ref.		Ref.	
College degree or above	303	64.7	165	35.3	1.41 (0.95–2.07)	0.085	1.60 (1.03–2.49)	0.036
Student								
No	301	62.7	179	37.3	Ref.			
Yes	79	63.7	45	36.3	1.04 (0.69–1.58)	0.837		
>6,000	146	58.4	104	41.6	Ref.		Ref.	
≤6,000	234	66.1	120	33.9	1.39 (0.99–1.94)	0.054	1.86 (1.28–2.72)	0.001
Family relationships								
General/disharmonious	181	66.3	92	33.7	Ref.			
Harmonious	199	60.1	132	39.9	0.77 (0.55–1.07)	0.118		
Whether think that “anal sex with men” is more likely to be infected with HIV than “vaginal sex with women, anal sex with women, or oral sex with men”								
No	36	43.9	46	56.1	Ref.		Ref.	
Yes	344	65.9	178	34.1	2.47 (1.54–3.98)	<0.001	1.53 (0.89–2.63)	0.123
Whether believe that the “receptive partner” role is more susceptible to HIV infection than the “insertive partner” role								
No	132	55.5	106	44.5	Ref.		Ref.	
Yes	248	67.8	118	32.2	1.69 (1.21–2.37)	<0.001	1.23 (0.84–1.79)	0.286
Whether think using enhancers (such as Rush Popper) increases the risk of HIV infection								
No	95	50.5	93	49.5	Ref.		Ref.	
Yes	285	68.5	131	31.5	2.13 (1.49–3.03)	<0.001	1.64 (1.11–2.42)	0.013
Whether learned about HIV/AIDS or mpox information through online media platforms (such as official accounts, apps, etc.) in the past year								
No	21	30.4	48	69.6	Ref.		Ref.	
Yes	359	67.1	176	32.9	4.66 (2.74–8.18)	<0.001	2.84 (1.58–5.24)	<0.001
Whether received HIV/AIDS or mpox prevention services (including promotional materials and distribution of condoms, counseling services, training sessions, etc.) in the past year								
No	92	46.0	108	54.0	Ref.		Ref.	
Yes	288	71.3	116	28.7	2.91 (2.05–4.15)	<0.001	2.01 (1.36–2.99)	<0.001
Whether have a stable same sexual partner currently								
No	247	62.8	146	37.2	Ref.			
Yes	133	63.0	78	37.0	1.01 (0.71–1.43)	0.965		
Whether have anal sex with the same sexual partner in the last 6 months								
No	151	60.9	97	39.1	Ref.			
Yes	229	64.3	127	35.7	1.16 (0.83–1.62)	0.389		
Whether have ever engaged in one-night stands of same sexual activity								
No	192	57.5	142	42.5	Ref.		Ref.	
Yes	188	69.6	82	30.4	1.69 (1.21–2.38)	0.002	1.38 (0.95–2.01)	0.092
Whether have been tested for HIV in the last year?								
No	55	46.2	64	53.8	Ref.		Ref.	
Yes	325	67.0	160	33.0	2.36 (1.57–3.56)	<0.001	1.44 (0.90–2.28)	0.122

CNY, Chinese yuan.

### 3.2 Awareness of mpox prevention and control knowledge

Regarding the source of mpox infection, most participants (75.66%; 457/604), knew that infected individuals were the sources of further infection. The top-three recognized transmission routes were sexual transmission (to be confirmed), close contact with patients, and droplet transmission. The highest number of participants believed that MSM were the most susceptible, accounting for 52.65% (318/604), followed by HIV-infected individuals at 43.21% (261/604). The three most recognized clinical manifestations of mpox were fever, skin rashes and lymphadenopathy. The top-three preventive measures were deemed avoiding areas with mpox outbreaks, having protected sex with regular partners, and vaccination. The overall mpox awareness rate was 62.91% (380/604). Table 2 presents these results.

**Table 2:** Knowledge and awareness of mpox among participants.

Variables	Total (*n* = 604)	
	*n*	%
Sources of mpox		
Infected animals	420	69.54
Infected people	457	75.66
Unclear	145	24.01
Transmission routes of mpox		
Close contact with patients	416	68.87
Droplet transmission	342	56.62
Contact with contaminated items	295	48.84
Vertical transmission	204	33.77
Sexual transmission (to be confirmed)	447	74.01
Unclear	130	21.52
The people susceptible to mpox		
All	305	50.50
People who were not vaccinated against smallpox	187	30.96
MSM	318	52.65
People living with HIV	261	43.21
Unclear	116	19.21
Clinical symptoms		
Chills and fever	334	55.30
Enlarged lymph nodes	327	54.14
Rash on the genitals or perianal areas or other parts of body	372	61.59
Weak	264	43.71
Muscle pain	265	43.87
Headache	225	37.25
Unclear	226	37.42
Preventive measure of mpox		
Stay away from the outbreak areas	445	73.68
Vaccination	381	63.08
Wearing mask	335	55.46
Hand disinfection	321	53.15
Regular sex partner and condom use	421	69.70
Unclear	131	21.69

### 3.3 Factors associated with awareness of mpox prevention and control

Univariate logistic regression analysis indicated statistically significant differences (*p* < 0.05) in the awareness of mpox prevention and control among participants according to different age groups, educational levels, and average monthly income. Additionally, awareness differed with beliefs regarding susceptibility to HIV through male anal sex as compared to other sexual activities, perceptions of HIV infection risk based on sexual roles, and beliefs about the risk of HIV infection with the use of enhancers (refer to drugs or substances that can enhance or promote sexual desire and behavior, such as Rush Poppers). Furthermore, significant differences in awareness were found according to recent (within the past year) exposure to HIV/AIDS or mpox information through online media platforms (such as public accounts, apps), recent receipt of HIV/AIDS or mpox prevention services (such as distribution of promotional materials and condoms, counseling services, training workshops), engagement in casual same sex encounters, and recent HIV testing.

Variables yielding *p* < 0.1 in the univariate regression analysis were included in multivariate logistic regression analysis to identify factors associated with awareness of mpox treatment and control measures. The analysis results showed that, compared to participants aged ≤29 years, the likelihood of awareness of mpox-related knowledge was increased by 65% for participants aged ≥30 years (adjusted odds ratio [aOR]: 1.65; 95% confidence interval [CI]: 1.10–2.51). Participants with a college degree or higher had a 60% increased likelihood of such awareness (aOR: 1.60; 95%CI: 1.03–2.49). Participants with a monthly income ≤6,000 CNY had an 86% increased likelihood of awareness (aOR: 1.86; 95%CI: 1.28–2.72). Participants who believed that using enhancers (such as Rush Poppers) increased the risk of HIV infection, had a 64% increased likelihood of awareness of mpox-related knowledge (aOR: 1.64; 95%CI: 1.11–2.42). Participants who had obtained HIV/AIDS or mpox information through online media platforms in the past year had an 84% increased likelihood of having awareness about mpox treatment or control (aOR: 1.84; 95%CI: 1.58–5.24). Participants who received HIV/AIDS or mpox prevention services in the past year had a 101% increased likelihood of awareness (aOR: 2.01; 95%CI: 1.36–2.99). The results of this analysis are presented in Table 1.

## 4 Discussion

In recent years, the emergence of mpox outbreaks within MSM populations across various countries and regions has garnered significant attention (23, 24). This investigation revealed a notable prevalence of risky sexual behaviors within the MSM community, coupled with a relatively modest level of awareness of mpox prevention and control measures within the study cohort (75.66%). This underscores the susceptibility of this population to mpox infection. To address this issue, educational campaigns and awareness initiatives that focus on the clinical manifestations and preventive strategies related to mpox among vulnerable populations, including MSM groups and travelers, must be intensified. Such efforts should aim to facilitate proactive self-health monitoring among at-risk individuals. The meta-analysis results showed that the pooled willingness rate of vaccinate against mpox among MSM was 58.5–77.0% (25, 26). MSM and people living with HIV demonstrate higher willingness to receive mpox vaccination due to their elevated risk of severe outcomes following infection (27, 28). However, vaccine hesitancy persists in the general population, primarily driven by concerns about potential adverse effects (29), the meta-analysis results showed that the prevalence of mpox vaccine hesitancy was 41.5% (26). To address this, public health strategies could employ analogy-based education campaigns (such as modeled after successful HPV vaccine cancer prevention messaging) to improve vaccine literacy.

The findings of this study suggested that individuals aged ≥30 years showed a 65% (aOR: 1.65; 95% CI: 1.10–2.5) higher likelihood of being aware of mpox-related information than were those aged ≤29 years. Older individuals tend to prioritize health concerns and demonstrate a proactive attitude towards seeking information on diseases such as mpox, indicating a greater willingness to acquire pertinent knowledge, including insights into diseases such as mpox (30). Health education initiatives are recommended to focus more extensively on delivering detailed and comprehensive knowledge about conditions such as mpox to younger age groups, aligning with their cognitive and attentional requirements relative to health issues.

Participants with higher education levels were likely to demonstrate a greater capacity and inclination to acquire and explore new knowledge actively (31). The findings of this study revealed that individuals with a college degree or above exhibited a 60% (aOR: 1.60; 95%CI: 1.03–2.49) higher likelihood of being informed about mpox. Those with advanced educational backgrounds typically have access to diverse information channels, facilitating their exposure to health-related knowledge through educational, occupational, and social avenues, encompassing insights into conditions, such as mpox, HIV, and COVID-19 (32). Conversely, individuals with at least a college degree may be able to comprehend and integrate specialized information, fostering a profound understanding of health issues, such as mpox, and enhancing their grasp of relevant details. Health education initiatives should target individuals with lower educational attainment levels by disseminating promotional materials on mpox-related information through multiple platforms, such as schools and workplaces, catering to their health knowledge needs and proactive information-seeking tendencies.

Given the potentially substantial impact of healthcare costs on financial well-being, this demographic group exhibits a heightened propensity for actively seeking information on diseases, such as mpox (33). Findings from this study revealed that individuals earning an average monthly income ≤6,000 CNY were 86% (aOR: 1.86; 95%CI: 1.28–2.72) more likely to be knowledgeable about mpox, highlighting a deeper level of understanding among lower-income brackets regarding mpox-related information. Moreover, lower-income demographics may favor gathering health knowledge through platforms such as social media and public health initiatives, which resonate closely with their everyday lives and requirements, and effectively capture their attention.

The use of enhancers (such as Rush Poppers) during sexual activities may lead to increases in risky behaviors, such as engaging in unsafe practices that can result in issues such as HIV infection, prompting individuals to become more aware of related health issues, including diseases such as mpox (34). The findings of this study revealed that participants who perceived that the use of enhancers (such as Rush Poppers) could increase the risk of HIV infection were 64% (aOR: 1.64; 95%CI: 1.11–2.42) more likely to be informed about mpox-related details. This implies a potential correlation between awareness of the elevated HIV risk linked to enhancer use and the comprehension of diseases such as mpox. This awareness may mirror the participants’ health concerns and recognition of the health risks associated with certain sexual behaviors. However, individuals who recognize the relationship between enhancers (such as Rush Poppers) and health risks related to sexual activities may have a stronger inclination to seek health information actively, including gaining insights into conditions such as mpox. They are likely to be more vigilant about their health status and to possess a certain level of awareness and cautiousness regarding potential health risks (35). Future health education initiatives should target populations who are vulnerable to risks associated with enhancer use, emphasizing the dissemination and reinforcement of information regarding health risks associated with sexual behaviors and diseases, to enhance awareness and vigilance, thus mitigating potential health risks stemming from unsafe sexual practices.

Accessing information via the internet is a convenient and expeditious method for participants to access health-related information at anytime and anyplace, through platforms such as public accounts and apps that provide insights into various diseases (36). This ease of access increases the probability of participants’ proactively seeking and obtaining knowledge about diseases, such as mpox, thereby enriching their comprehension of mpox-related details. This study revealed that individuals who had accessed HIV/AIDS or mpox information through online media in the past year were 84% (aOR: 1.84; 95%CI: 1.58–5.24) more likely to be to have mpox-related information, underscoring the influential impact of retrieving information on HIV/AIDS or mpox via online platforms. Online media platforms facilitate the broad and immediate dissemination of information, including diverse health data and knowledge (37). When participants encounter information on conditions, such as HIV/AIDS or mpox, through online media, they may develop a heightened focus on health matters, and may be motivated to seek further knowledge on diseases such as mpox, actively. We recommend that disease-related content dissemination should be enhanced on online platforms, particularly via public accounts and apps, to provide accurate and detailed information regarding disease prevention and treatment. This strategy could attract more participants to acquire relevant knowledge proactively, thereby boosting their awareness and comprehension of diseases such as mpox.

MSM who receive preventive services often come into contact with informational materials, counseling services, and training seminars related to HIV/AIDS or mpox. These services and materials encompass knowledge of the causes, transmission routes, and prevention methods related to mpox, thereby enhancing participants’ awareness of this disease (38). The results of our study indicated that participants who had received preventive services in the past year, including the distribution of promotional materials and condoms, counseling services, and training seminars related to HIV/AIDS or mpox, had a 101% (aOR: 2.01; 95%CI: 1.36–2.99) increased likelihood of being aware of mpox-related knowledge. This suggests that individuals who received preventive services were more likely to have a deeper understanding of mpox-related information. Moreover, institutions providing preventive services often conduct targeted awareness campaigns and education specific to health issues, including diseases, such as HIV/AIDS and mpox. By participating in these services, individuals may acquire more structured and comprehensive health knowledge, contributing to an elevated level of awareness of mpox-related information (39). We recommend that preventive services related to diseases such as mpox, including promotional materials, counseling services, and training seminars, should continue to be offered and should be promoted, in order to enhance the understanding of MSM related to mpox. By engaging in these preventive services, MSM can enhance their self-protection and health awareness.

This study had several limitations. The cross-sectional design hindered the establishment of causal relationships by focusing solely on associations. A potential bias may have arisen because of potential sampling bias, generalizability issues, or social desirability bias in self-reported behaviors that influenced the results. Furthermore, epidemiological questionnaire surveys may result recall bias, compromising the accuracy of the results. The study utilized both univariate and multivariate logistic regression analyses to mitigate the confounding factors. Future investigations should incorporate larger sample sizes, prospective cohort studies, and intervention trials across multiple centers. This approach facilitates a deeper comprehension of causal relationships among associated factors, reduces confounding effects, and bolsters the credibility of the research outcomes. Increasing the sample size and adopting multicenter research designs in future could enhance the representativeness and applicability of the findings. Subsequent research phases should integrate qualitative research methods to explore participants’ attitudes, beliefs, and behaviors comprehensively, thereby further elucidating the factors influencing the transmission and prevention of mpox. By addressing these limitations and embracing comprehensive and forward-looking research designs and analytical techniques, a more profound understanding of mpox transmission and prevention can be established, thereby offering valuable guidance and support for public health policies and practices. Meanwhile, excessive focus is placed on socioeconomic and demographic variables (age, income, education) in this study, we will add structural or healthcare access-related factors in the future study, which may have equal or greater influence on awareness levels.

## 5 Conclusion

The study indicated that individuals who have lower education levels, who perceive enhancer use as increasing the risk of HIV infection, who seek information about mpox through online platforms, and who have received HIV or mpox preventive services, demonstrated a heightened awareness of mpox. These findings underscore the importance of reinforcing tailored awareness campaigns and educational initiatives, as well as preventive measures tailored to specific groups to improve the dissemination of mpox-related knowledge. Future endeavors should prioritize targeted promotional campaigns, augment the distribution of information concerning mpox through online channels, and expand the reach of mpox prevention services to ensure widespread mpox-related awareness and prevention measures within society.

## Data Availability

The original contributions presented in the study are included in the article/supplementary material, further inquiries can be directed to the corresponding author.

## Footnotes

^1^ https://www.r-project.org/
